# Cases of Crimean-Congo haemorrhagic fever in North Macedonia, July to August 2023

**DOI:** 10.2807/1560-7917.ES.2023.28.34.2300409

**Published:** 2023-08-24

**Authors:** Dejan Jakimovski, Krsto Grozdanovski, Goran Rangelov, Verica Pavleva, Pavle Banović, Alejandro Cabezas-Cruz, Katerina Spasovska

**Affiliations:** 1 Faculty of Medicine, Ss. Cyril and Methodius University in Skopje, Skopje, Republic of North Macedonia; 2 University Clinic for Infectious Diseases and Febrile Conditions, Skopje, Republic of North Macedonia; 3 Infectious Disease Department, Clinical Hospital Shtip, Shtip, Republic of North Macedonia; 4 Clinic for Lyme Borreliosis and Other Tick-Borne Diseases, Pasteur Institute Novi Sad, Novi Sad, Serbia; 5 Department of Microbiology with Parasitology and Immunology, Faculty of Medicine in Novi Sad, University of Novi Sad, Novi Sad, Serbia; 6 Anses, INRAE, Ecole Nationale Vétérinaire d’Alfort, UMR BIPAR, Laboratoire de Santé Animale, Maisons-Alfort, France; * These authors contributed equally to the work and share first authorship

**Keywords:** CCHF, CCHFV, *Hyalomma*, North Macedonia

## Abstract

The last report of Crimean-Congo haemorrhagic fever (CCHF) in North Macedonia was more than 50 years ago in the northwest. We report on a fatal CCHF case following a *Hyalomma* tick bite in the east of the country in July 2023. Tracing of 67 contacts identified CCHF in one healthcare worker (HCW) providing care for the patient. Monitoring of contacts is concluded (including further 11 HCW contacts), thus far 28 days after the death of the case no additional cases were identified.

Crimean-Congo haemorrhagic fever (CCHF) is a severe tick-borne disease caused by the CCHF virus from the Bunyaviridae family and included in the World Health Organization (WHO) list of important emerging infectious diseases with pandemic potential [1]. The last report of CCHF in North Macedonia was more than 50 years ago, when an outbreak occurred in a village located in Polog, in the northwestern region of the country. The distribution of the CCHFV is closely linked to the presence of *Hyalomma* tick species, which act as the main vectors for this pathogen [2]. We describe a fatal case of autochthonous CCHF (Case 1) in North Macedonia as well as a nosocomial infection in a healthcare worker who cared for the patient (Case 2) and preliminary results from tracing of contacts.

## Case 1

**Figure 1:**
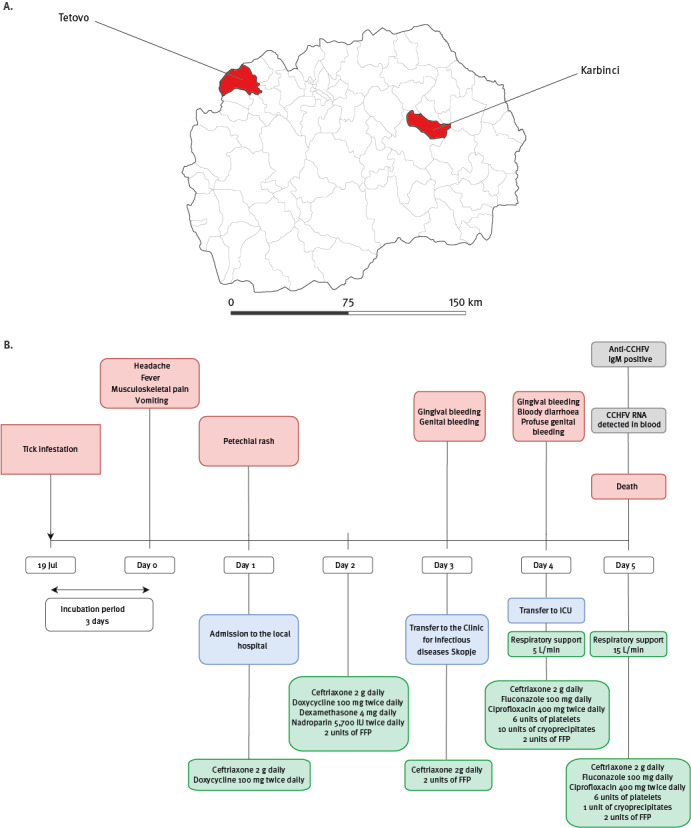
Geographical location of the Crimean-Congo haemorrhagic fever case (Case 1) and a previous case (A) and timeline of Case 1 (B), North Macedonia, July 2023

CCHFV: Crimean-Congo haemorrhagic fever virus; FFP: fresh frozen plasma; ICU: intensive care unit; IU: international unit.

On 25 July 2023, a patient in their late twenties with no previous medical history was admitted to the Clinic for Infectious diseases in Skopje, North Macedonia. They had earlier presented at a local hospital with a fever (38.6°C), rash, musculoskeletal pain, headache and vomiting, which began 3 days after a tick bite. The patient (Case 1) lived in a village located in a rural area of the eastern region of North Macedonia (Figure 1A) and had not travelled outside that region in the preceding month. They noticed a tick bite on 19 July on the lower left abdomen, 1 hour after they had visited a tobacco field, and the tick was promptly removed (Figure 1B). No similar symptoms were reported in any other household members.

Day 0 is the day of symptom onset, the following days are days after symptom onset.

The North Macedonia shapefile for mapping at district and municipality levels is available at the GADM database of Global Administrative Areas (v2.8, https://gadm.org/). The map was generated by using QGIS v3.12 (QGIS Development Team, 2020). Timeline generated using open-source software draw.io (https://app.diagrams.net/).

In the upper part of the timeline, the most significant signs and symptoms are presented while the most important interventions are presented in the lower part.

The symptoms had begun 3 days after the tick bite (Figure 1B). On Day 1 after symptom onset, the patient consulted a physician at the local hospital and was admitted for suspected rickettsiosis based on the symptoms and the history of the tick bite. They received antibiotic therapy with ceftriaxone 2 g daily intravenously (i.v.) and doxycycline 100 mg twice daily orally (p.o.). On the same day, a petechial rash was noted and initial laboratory investigations revealed severe leucopenia and thrombocytopenia accompanied by significantly elevated D-dimer and secondary activated fibrinolysis (Table 1). Brain and thorax computer tomography, as well as abdominopelvic ultrasonography, showed no abnormalities. The treatment was further supplemented with nadroparin 5,700 international units (IU) twice daily, 2 units of fresh frozen plasma (FFP) and dexamethasone 4 mg daily. Nevertheless, the patient remained febrile and the thrombocyte counts continued to decline (Table 1). On Day 3 after symptom onset, bloody discharge from the genitals was observed and the patient was transferred to the Clinic for Infectious Diseases in Skopje, North Macedonia (CID Skopje).

**TABLE 1:** Summary of the most important laboratory findings of Case 1 of Crimean-Congo haemorrhagic fever, North Macedonia, July 2023

Parameters and assays	Day 1 after symptom onset	Day 3 after symptom onset	Day 4 after symptom onset	Day 5 after symptom onset	Reference values
**Biochemical parameters**					
Aspartate aminotransferase (U/L)	56	218	726	6,530	10–47
Alanine transaminase (U/L)	15	39	162	1150	10–52
Creatine kinase (U/L)	NA	932	756	392	30–170
Lactate dehydrogenase (IU/mL)	370	919	1,254	5,948	120–246
C-reactive protein (mg/L)	72	7	12	42	0–10
**Haematological parameters**					
Haemoglobin (g/L)	134	130	120	105	110–160
Leukocytes (10^9^/L)	2.29	1.13	1.1	2.6	4–10
Platelets (10^9^/L)	77	24	11	9	150–400
Lymphocytes (%)	11	43	29	44	21–25
Red blood cells (10^12^/L)	6.18	6.61	6.16	5.3	3.8–5.8
D-dimer (ng/mL)	21,620	8,160	NA	3,713	0–500
Prothrombin time (s)	15	14.7	NA	22.9	9.8–14.2
Thrombin time (s)	19.1	0	NA	120	16.1–24.1
INR	1.51	1.48	NA	2	0.8–1.2
**Microbiological analyses**					
Anti-CCHFV IgM	NA	NA	NA	Positive	Not applicable
CCHFV RNA	NA	NA	NA	Positive	Not applicable

CCHFV: Crimean-Congo haemorrhagic fever virus; INR: international normalised ratio; NA: not analysed; U: unit.

**Figure 2:**
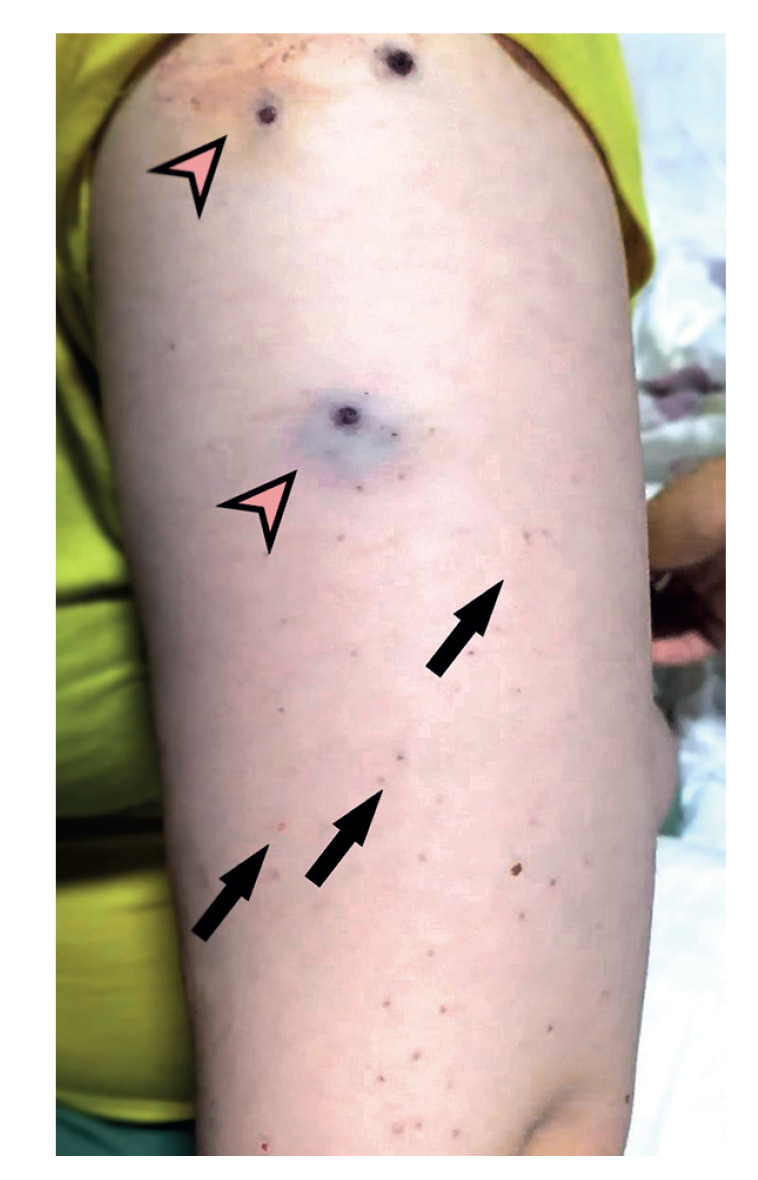
Skin lesions of Case 1 of Crimean-Congo haemorrhagic fever on Day 3 after symptom onset, North Macedonia, July 2023

Upon admission on Day 3 after symptom onset, the patient was conscious, febrile, hypotensive and bradycardic. Bruising on the left upper arm was present (from low-molecular-weight heparin administration), and a petechial rash was found on her back and extremities (Figure 2). The initial blood analysis showed severe leucopenia and thrombocytopenia (Table 1). Additionally, increased aminotransferase activity, lactate dehydrogenase, creatinine kinase and coagulopathy were noted. Treatment with ceftriaxone 2 g daily i.v., ciprofloxacin 400 mg twice a day i.v., fluconazole 100 mg p.o. daily was initiated and patient received 2 units of FFP to replenish coagulation factors (Figure 1B).

Black arrows point towards petechial bleeding. Arrow heads indicate haematomas caused by the administration of low-molecular-weight heparin and phlebotomy.

On Day 4 after symptom onset, the patient developed signs of respiratory failure and was transferred to the intensive care unit, where she received oxygen support at 5 L per minute. Their condition worsened with persistent fever, hypotension, tachycardia, dyspnoea and development of a haemorrhagic syndrome, manifested by bloody diarrhoea and profuse genital bleeding. In response to the clinical condition, the patient was administered 2 units of FFP, 6 units of thrombocytes and 10 units of cryoprecipitates. A wide spectrum of microbiological investigations was conducted but yielded no significant findings.

On Day 5 after symptom onset, respiratory failure worsened, requiring increased oxygen support. The patient also experienced severe hepatic insufficiency, oliguria, exacerbated coagulopathy and hypotension with need of vasopressor stimulation. Due to the recent tick bite history and the clinical presentation, a suspicion of CCHF emerged as a potential cause. Additionally, photographs of different tick genera known to be present in North Macedonia (*Ixodes, Ripicephalus, Dermacentor, Haemaphysalis, Hyalomma*) allowed the patient’s relative to identify the *Hyalomma* tick genus as the one removed from the patient’s skin, based on the specific size and banded legs of the tick.

The patient tested positive for anti-CCHFV IgM (CCHFV Mosaic 2 IgM indirect immunofluorescence tests; Euroimmun, Germany) in serum at dilution 1:10, and CCHFV was directly detected in the blood via the Viasure Crimean-Congo haemorrhagic Fever Virus Real-Time PCR Detection Kit using CFX96 Touch Real-Time PCR Detection System (Bio-Rad Laboratories, United States) on Day 5 after symptom onset. On the same day, they experienced a sudden onset of seizures and, shortly after, went into cardiac arrest. Cardiopulmonary resuscitation was diligently administered, but unfortunately, the outcome was fatal.

## Public health measures and results of tracing contacts of Case 1

**Figure 3:**
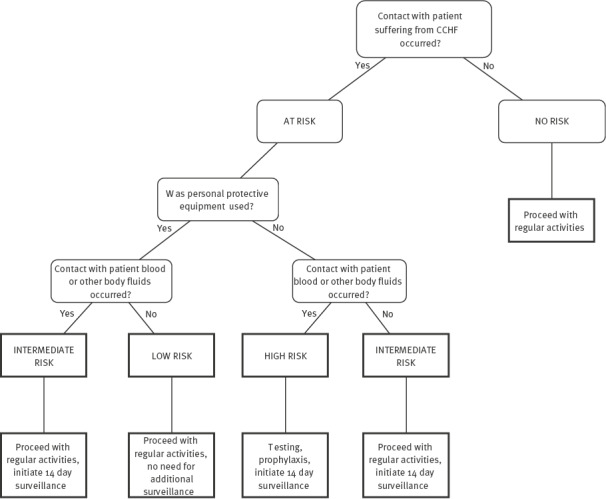
Decision tree for assessing risk of Crimean-Congo haemorrhagic fever infection for contacts of cases, North Macedonia, July-August 2023

Public health authorities were informed immediately upon receipt of the CCHF diagnosis, and contact tracing was started the morning after the diagnosis and fatal outcome of Case 1. Contact tracing was conducted for household members and healthcare workers (HCW) in all hospitals where the patient stayed, and appropriate disease risk classifications were assigned (Figure 3). The WHO and the European Centre for Disease Prevention and Control (ECDC) were informed promptly the next day. Of the 67 identified contacts of Case 1, five were assessed as high, 24 as medium and 38 as low risk. Contacts with high and medium risk were put on self-monitoring for the next 14 days. They were advised to continue their daily activities, to monitor their body temperature twice a day and to report if they experience any symptoms. Following the finalisation of contact tracing procedures and writing the public health authorities' report, the general public was informed via various media outlets on 30 July.

CCHFV: Crimean-Congo haemorrhagic fever virus.

## Case 2

Among the 24 contacts at medium risk, a HCW in their late fourties who was working as a hospital attendant with the patient on Day 5 after their symptom onset, showed symptoms of infection on Day 8 after exposure. Immediate testing yielded a positive PCR finding for CCHFV in blood and hospitalisation in the intensive care unit ensued. Although the HCW was wearing personal protective equipment (PPE) including gloves, mask, apron and face shield while taking care of the patient, the exposure could have occurred during possible improper removal of the PPE.

Contact tracing was performed once again, revealing six low-risk and 11 medium-risk contacts: 11 of these were subsequently placed under self-monitoring, bringing the total number of identified contacts to 84 (including Case 2) of whom 40 were put on self-monitoring.

Initial symptoms of Case 2 were elevated temperature (38.5°C) and conjunctivitis. On Day 2 of their hospitalisation, reduction of platelet count was detected as well as increased levels of lactate dehydrogenase and creatine kinase (Table 2). On Day 7 of their hospitalisation, antiviral intravenous medication with ribavirin was introduced according to Médecins sans Frontières Guideline for Management of Crimean Congo Haemorrhagic Fever [3]. Defervescence occurred the same day and the HCW remained afebrile until hospital discharge. Levels of lactate dehydrogenase and creatine kinase reduced on Day 8 of the hospitalisation, as predictors of a favourable outcome (Table 2). On Day 14, the platelet count had returned to normal and enzyme levels had nearly reverted to their baseline values. However, there was an observed decrease in red blood cell count and haemoglobin levels, which was likely attributed to the inclusion of ribavirin in the therapy protocol and its associated side effects. Case 2 was discharged on Day 18 in stable condition.

**TABLE 2:** Summary of the most important laboratory findings of Case 2 of Crimean-Congo haemorrhagic fever, North Macedonia, July–August 2023

Parameters and assays	Symptom onset Day 8 after exposure to Case 1	Day 2 after symptom onset	Day 4 after symptom onset	Day 8 after symptom onset	Day 13 after symptom onset	Reference values
Biochemical parameters						
Aspartate aminotransferase (U/L)	24	70	515	329	23	10–47
Alanine transaminase (U/L)	15	32	269	229	58	10–52
Creatine kinase (U/L)	144	720	621	182	50	30–170
Lactate dehydrogenase (IU/mL)	183	269	721	460	285	120–246
C-reactive protein (mg/L)	3	2	12	15	49	0–10
Haematological parameters						
Haemoglobin (g/L)	124	123	133	117	87	110–160
Leukocytes (10^9^/L)	3.3	2.6	5.0	5.3	7.5	4–10
Platelets (10^9^/L)	141	89	51	62	168	150–400
Lymphocytes (%)	10	19	34	26	10	21–25
Red blood cells (10^12^/L)	4.6	4.7	5.0	4.5	3.3	3.8–5.8
D-dimer (ng/mL)	1,199	1,092	7,822	775	2,320	0–500
Prothrombin time (s)	13.2	11.1	11.9	10.3	15.3	9.8–14.2
Thrombin time (s)	19.9	21.7	45.8	31.9	31	16.1–24.1
Microbiological analyses						
CCHFV RNA	Positive	NA	NA	NA	NA	Not applicable

CCHFV: Crimean-Congo haemorrhagic fever virus; NA: not analysed; U: unit.

## Discussion

We describe two cases of CCHF: Case 1 in a rural area of the eastern part of North Macedonia and a secondary case, Case 2, a healthcare worker. Contact monitoring is concluded, thus far 28 days after the death of Case 1 no additional cases have been identified among the 83 contacts. A third patient with CCHF from another region of the country, with no epidemiological link to the confirmed cases or identified contacts from contact tracing, was admitted to our clinic. This could signal a potential outbreak of CCHF in the country, or it could demonstrate an increased awareness among medical professionals on a previously overlooked disease.

Currently in Europe, there are no approved vaccines or specific therapies for CCHF [4]. The first outbreak of CCHF in North Macedonia was reported in the summer of 1979, in a village near Tetovo city, with 13 members of a family contracting the disease and two of them tragically passing away. The infection likely occurred due to contact with blood from a man in their sixties who had been bitten by *Hyalomma* ticks and for whom the infected family members had provided care [5].

Subsequently, CCHF cases were mainly reported in neighbouring Kosovo*: two cases in 1989 and followed by two cases in 1993 and outbreaks comprising 45 cases in 1995, eight cases in 1996 and seven in 1997 [5]. Central Serbia also reported one case in 1999 from a person who acquired the infection while staying in Kosovo [6]. Infections occurred between March and September, and all affected individuals were living in rural areas where animal husbandry and agriculture were their main source of income [5]. Between 2013 and 2016, there were 32 reported cases of CCHF in Kosovo. The cases were from central and south-western parts of Kosovo, near Albania and North Macedonia [7]. In Albania, the most recent cases of CCHF occurred between 2003 and 2006, with 13 patients from the Kukës district [8]. Notably, this district is ca 75 km from the North Macedonian village where Case 1 lived, however, separated by a massive mountainous range.

CCHF has also been reported in Bulgaria, with evidence of CCHFV spreading westward, towards North Macedonia [9]. In 2008, a CCHF case occurred in Bulgarian town of Goce Delchev, which is ca 100 km from the village where Case 1 lived [10].

Since 2015, CCHF has been included in the WHO list of the most important emerging infectious diseases with pandemic potential [1]. The virus is circulating among small ruminants in Bosnia and Hercegovina and Romania [11,12]. Additionally, there are reports of the emergence of *Hyalomma marginatum* and *Hyalomma rufipes*, ticks known to carry CCHFV in Hungary, along with human exposure to the virus [13,14].

However, data on the prevalence of the main vector for CCHFV, the *Hyalomma* tick species, in North Macedonia are scarce. *Hyalomma* ticks have been detected in Skopje, north-eastern and south-eastern regions [15], with the highest seroprevalence of anti-CCHFV found in cattle from the region bordering Serbia and Bulgaria. Unfortunately, this survey did not include the eastern region, where the CCHFV infection occurred in the patient described here.

## Conclusion

Considering that CCHF is a neglected disease with pandemic potential, it seems essential to conduct a risk assessment for the population living in the area where the case occurred. Additionally, a seroprevalence study of the local population and surrounding areas, as well as tick sampling for the presence of CCHFV in the same region would be needed to determine the level of exposure to CCHFV in the population and the prevalence of tick infections. Furthermore, we propose promoting cross-border cooperation among the Balkan countries to standardise diagnostic procedures and raise awareness among physicians. This involves building diagnostic capacity and networks for sharing information about CCHFV cases. By doing so, we can effectively plan preventive measures and assess the impact of this disease on the population of Balkan countries possibly affected by CCHFV.

## *Note

This designation is without prejudice to positions on status, and is in line with United Nations Security Council Resolution 1244/99 and the International Court of Justice Opinion on the Kosovo Declaration of Independence.
