# Addendum for Euro Surveill. 2023;28(34)

**DOI:** 10.2807/1560-7917.ES.2023.28.41.231012a

**Published:** 2023-10-12

**Authors:** Eurosurveillance editorial team

**Affiliations:** 1 European Centre for Disease Prevention and Control (ECDC), Stockholm, Sweden

In the article entitled ‘Cases of Crimean-Congo haemorrhagic fever in North Macedonia, July to August 2023’ by Jakimovski et al., published on 24 August 2023, the following additions were made on 12 October 2023:

The manuscript now specifies the teams that performed the contact tracing for Cases 1 and 2; a citation and a personal communication note have been added for information obtained about the cases. 

Information about the preparation of Figure 3 was added under the figure.

The laboratory that did the CCHFV analysis has been named, in the text and in the footnote of the relevant figure.

It has been noted that information on tick sampling was not available to the authors at the time of writing. 

An Acknowledgement section has been added.

